# 

**Published:** 1910-01-08

**Authors:** 


### BOOKS RECEIVED.

Eyre and Spottiswoode.

"Pears' Annual."

Bailliere, Tindall, and Cox.

"Quack Remedies." By D. Walsh, M.D.

"Pulmonary Tuberculosis and Sanatorium Treatment." By C. Muthu, M.D., etc.

Henry J. Glaisher.

"Soured Milk and Pure Cultures of Lactic Acid Bacilli in the Treatment of Disease." By George Herschell, M.D.

Butterworth and Co.

"The Law Relating to Poisons and Pharmacy, with Notes and Cases." By W. S. Glyn-Jones.

### Gifts and Bequests.

Sir Benjamin L. Cohen, who left estate valued at £385,282 2s., left £200 to the Jews' Hospital and Orphan Asylum; £300 to St. Bartholomew's Hospital; and £100 each to St. Mary's Hospital, Paddington, the Hospital for Consumption, Brompton, the London Homœopathic Hospital, the Royal Hospital for Incurables, Putney, the Mount Vernon (Hampstead) Hospital for Consumption, the Great Northern Central Hospital, Holloway Road, and the East London Hospital for Children and Dispensary for Women, Shadwell.

The Covent Garden Committee of Charing Cross Hospital, instituted to collect funds from the traders in Covent Garden Market, has paid over the sum of £300, which is £53 more than in 1908.

Mrs. Florence Elizabeth McMillan has left £100 to the Royal Hospital for Incurables at Putney.

Mrs. Ellen Constantia Barker, after various bequests, has left two-ninths of the ultimate residue to the Royal Hospital for Incurables at Putney.

The Hon. Secretaries of King Edward's Hospital Fund for London have received from the League of Mercy, as its contribution for the year 1909, £19,000.

The Royal Berkshire Hospital at Reading was reported to have received from the late Miss Morrison £5,000, free of legacy duty.

### THE QUESTION BOX.

**SPECIAL NOTICE.**—Questions of interest affecting the honorary medical staff and hospital administration in all departments are invited. When any point arises we hope our readers will formulate it in a question and so co-operate to make this column of real value and interest. In this way the experience of the best-managed hospitals should be made helpful to the whole hospital world. We shall welcome the contribution of both questions and answers.

N.B.—The publication of an answer in this column does not necessarily preclude the publication of subsequent answers to the same question, for a question may involve points which require to be threshed out and fully discussed. Answers, if published, will be paid for at scale rates.

### WOMEN ON HOSPITAL BOARDS.

Question: Is it desirable that women should be appointed as members of the Governing and Executive Committees of voluntary hospitals?

### A London View.

By Conrad W. Thies, Secretary, Royal Free Hospital.

For many years past women have amply proved their capacity and fitness to serve on public boards. At the present time more than one thousand women are acting as Poor Law Guardians, while many hundreds are efficiently discharging responsible public duties as school board managers, medical officers, factory inspectors, etc., and large numbers hold other responsible positions throughout the country and have proved themselves thoroughly efficient for such work. In the face of these facts it is somewhat difficult to account for the reluctance which has been shown by a large majority of the committees of voluntary hospitals and kindred institutions to admit women to a share of their administrative work. Those hospitals which have already admitted women to seats on their management have found them most valuable members of their administrative bodies. To mention only a few of these institutions I may name the Royal Infirmary, Edinburgh; the Western Infirmary, Glasgow; the Victoria Hospital, Belfast; Addenbrooke's Hospital, Cambridge; and the Royal Free Hospital, London. The qualifications required of women to serve on hospital committees are much the same as for men. Women of the right kind bring special qualifications for such work, namely, sympathy and enthusiasm, together with a practical knowledge of domestic economy. The internal arrangements of a hospital are much the same as that of any well-ordered household and the same kind of knowledge and practical experience are essential to good administration. Such questions as dietary, linen, and domestic supplies, laundry work, and the supervision of large staffs of nurses and domestic servants are all essentially women's work, and their advice thereon is invaluable to the administration. We are constantly being told that in these days "taxation without representation is tyranny." Surely this argument may be fitly applied to this question, for an analysis of the contributors to our voluntary charities will show that a very large proportion of the donors are women.

### A Cardiff View.

Answer: Yes.

By Leonard D. Rea, Secretary and General Superintendent, Cardiff Infirmary.

Under the rules of this hospital women are eligible for election or appointment upon its committees, in just the same way as men are eligible, and the practice, which has been in vogue for many years, has worked admirably. A number of ladies are members of the board of management (the chief executive committee), whilst several serve on the house and nursing committees, but so far the finance and surgery and dispensary committees have not appealed to feminine ambition. The influence of these lady members has been consistently for good, their womanly feeling leavening the harsher judgment of men where questions arise when opportunities for greater usefulness in the relief of suffering do not commend themselves to commercial experience. They are also of great service in matters coming specially within a woman's province, and even in the selection of tenders for articles in which a woman's judgment may prudently be consulted. A further great advantage lies in the fact that their practical knowledge of the conduct of the hospital enables lady members to speak with authority, when its management may, within their knowledge, be unjustly criticised, and ladies of social influence, thus equipped, are powerful in establishing a feeling of confidence in the minds of their friends, which finds its natural expression in maintaining the credit of the institution at the bankers.

### COAL DELIVERIES AND THEIR WEIGHT.

Question: What percentage of reduction in the coal bill of an institution may be expected from weighing coal on delivery before accepting it?

### A Cardiff Experience.

By Leonard D. Rea, Secretary and General Superintendent, Cardiff Infirmary.

It is, of course, as important to weigh coal on delivery as it is to weigh any other commodity, but it is sometimes difficult to arrange for the provision of a weighbridge. Failing the latter, a good plan is to deal direct with a colliery, or insist upon the contractor securing that the colliery invoice, as to weight of coal in each truck, is forwarded direct to the hospital. In this district one must accept the colliery weight, and my experience is that it rarely differs from the delivery weight (as ascertained at the public weighbridge) by more than half a hundred weight or so in a ten-ton truck, which quantity may easily be lost in transit. Sometimes it is curious to find that the weight on delivery is even greater than the weight certified at the colliery. This doubtless results from rain getting into the truck or a discrepancy in the tare of the wagon. When the arrangement obtains that the colliery weights are to be accepted (save in the event of some gross disparity) as the basis of payment, the public weighbridge may perhaps be regarded as an efficient check, but a weighbridge belonging to the institution is preferable, and may be used for other things besides coal. Where a leakage exists, it should also be borne in mind that, not only are you paying for a proportion of coal not delivered, but also for the hauling or cartage of the same.

By A Medical Superintendent of an Institution.

Some inquiries I made of a firm of weighing-machine makers elicited that one might expect a saving of at least a hundredweight per ton—*i.e.* 5 per cent. The price mentioned—in my answer published in your December 25 issue—for a weighbridge (£10) must, however, mean a second-hand one, which this firm would not recommend as it would need repairs about every two years. A new machine should not want attention for five years, and would cost about £19, the price varying a little with the railway carriage.

THE
### GRADUATES' MEDICAL SCHOOLS.

DIARY FOR THE WEEK, JANUARY 10 to 14.

### ST. JOHN'S HOSPITAL FOR DISEASES OF THE SKIN, Leicester Square, W.C.

At 6 p.m.—Jan. 13, Chesterfield Lecture: **Eczema** (Varieties, Symptoms, Treatment).

### THE BROMPTON HOSPITAL FOR CONSUMPTION AND DISEASES OF THE CHEST, Fulham Road, S.W.

At 4 p.m.—Jan. 12, Sir R. D. Powell, Bart., K.C.V.O., **Introductory.**

### MEDICAL GRADUATES' COLLEGE AND POLYCLINIC, 22 Chenies Street, W.C.

At 4 p.m.—Jan. 10, Dr. J. E. R. McDonagh, **Clinique (Skin).**
At 5.15 p.m.—Jan. 10, Mr. Sydney Stephenson, **Ophthalmia Neonatorum.**
At 4 p.m.—Jan. 11, Dr. Lewis Smith, **Clinique (Medical).**
At 5.15 p.m.—Jan. 11, Mr. H. Stansfield Collier, **Post-Operative Treatment of Abdominal Cases.**
At 4 p.m.—Jan. 12, Mr. Arthur Edmunds, **Clinique (Surgical).**
At 5.15 p.m.—Jan. 12, Dr. Scanes Spicer, **A New Cardinal Principle in the Treatment of Disease and its Application in Disorders of the Nose and Throat.**
At 4 p.m.—Jan. 13, Sir Jonathan Hutchinson, **Clinique (Surgical).**
At 5.15 p.m.—Jan. 13, Dr. J. E. Squire, **Asthma and its Treatment.**
At 4 p.m.—Jan. 14, Dr. William Hill, **Clinique (Ear, Nose, and Throat).**

### THE POST-GRADUATE COLLEGE, West London Hospital, Hammersmith, W.

At 10 a.m.—Jan. 10 and 13, **Demonstration of Cases by Surgical Registrar.**
At 10 a.m.—Jan. 14, **Demonstration of Cases by Medical Registrar.**
At 12 noon.—Jan. 13, Dr. Bernstein, **Pathological Demonstration.**
At 12.15 p.m.—Jan. 11, Dr. Pritchard, **Practical Medicine.**
At 12.15 p.m.—Jan. 12, Dr. Grainger Stewart, **Practical Medicine.**
At 5 p.m.—Jan. 10, Dr. Morton, **X-Ray Examination of the Thorax.**
At 5 p.m.—Jan. 11, Dr. Saunders, **Clinical Lecture, with Cases.**
At 5 p.m.—Jan. 12, Dr. Beddard, **Medicine: Lecture I.**
At 5 p.m.—Jan. 13, Mr. Armour, **Thecal Abscess.**
At 5 p.m.—Jan. 14, Dr. Low, **Plague.**

### THE WEST-END HOSPITAL FOR NERVOUS DISEASES, Welbeck Street, W.

**Clinical Demonstrations for Practitioners and Senior Students.**

At 3 p.m., Mondays, Dr. Harry Campbell.
At 5.30 p.m., Tuesdays, Dr. James Mackenzie.
At 3 p.m., Wednesdays, Dr. F. S. Palmer.
At 3 p.m., Thursdays, Dr. T. D. Savill.
At 2 p.m., Fridays, Dr. Purves Stewart.
At 5.30 p.m., Fridays, Dr. E. D. Macnamara.

### ROYAL SOCIETY OF MEDICINE, 20 Hanover Square, W.

At 5.30 p.m.—Jan. 11, **Medical and Surgical Sections:**
*Adjourned joint debate* on "The diagnosis and treatment of duodenal ulcer." The following will take part: Dr. R. Hutchison, Dr. R. H. Hodgson, Dr. F. Craven Moore; Messrs. L. A. Bidwell, C. P. Childe, H. J. Paterson, Harold Collinson, B. G. Moynihan, and others.

At 7.45 p.m.—Jan. 13, **Obstetrical and Gynæcological Section:**
*Specimens.*—Mr. H. J. Paterson: Sarcoma of the uterus. Dr. J. B. Hellier: Adenoma malignum cysticum cervicis uteri. Dr. Cuthbert Lockyer: Two cases of pelvic hæmatocele of ovarian origin. Dr. Macnaughton-Jones: A specimen.
*Short Communication.*—Dr. Inglis Parsons: Notes on a case of prolapsus uteri with severe cardiac disease, operated on under local and spinal analgesia.
*Paper.*—Mrs. Scharlieb: "The relative frequency of malignancy in ovarian tumours."

At 5 p.m.—Jan. 14, **General Meeting of Fellows:** Election of candidates for Fellowship. **At 8 p.m., Clinical Section:**
*Cases.*—Dr. Howard Heaton: Rectal stricture treated by fibrolysia. Mr. J. E. Adams: Empyema cured by thoracoplasty. Mr. Herbert Tilley: Objective and rhythmical "clicking" noise in the left ear, associated with synchronous contraction of certain muscles forming the floor of the mouth. Mr. Somerville Hastings: Bending of the bones of both legs, and partial ankylosis of both hips. Dr. Parkes Weber: (1) Chronic arteritis obliterans of a lower extremity associated with phlebitis. (2) Intermittent claudication of the lower extremity due to arteritis obliterans (in a tobacconist). And other cases.

### APPOINTMENTS VACANT.

ST. JOHN'S HOSPITAL FOR DISEASES OF THE SKIN.—Honorary anæsthetist.
LONDON HOMŒOPATHIC HOSPITAL.—Assistant physician.
ROYAL ALBERT HOSPITAL, DEVONPORT.—Resident Medical officer.
SEAMEN'S HOSPITAL SOCIETY.—Assistant physician, assistant surgeon.

WE commend to the Local Education Authorities the example of Charlottenburg, remarks the Berlin correspondent of the *Journal of Education*. Charlottenburg, not content with inspection, has called woman to its succour. For four *Gemeindeschulen* it has appointed a *Schulschwester*. Her duties are to visit parents and require them to carry out the doctor's instructions; to take the sick child to the doctor when the mother cannot accompany him; to procure the parents' consent to small operations; to buy spectacles, bandages, trusses, etc.; in cases of malnutrition, uncleanliness, or insufficient clothing, to report on the financial position of the father; and to admonish the mother as to the importance of washing, bathing, and pure air. This new appointment has already justified itself so well that the medical officer recommends a like arrangement in the twenty-two other *Gemeindeschulen*.

THE HOSPITAL JANUARY 8, 1910.

*Name* ..................................

*Address* ..................................

..................................

**This Coupon must accompany manuscript or contributions intended for THE HOSPITAL.**

